# Coinfection With *Trypanosoma brucei* Confers Protection Against Cutaneous Leishmaniasis

**DOI:** 10.3389/fimmu.2018.02855

**Published:** 2018-12-11

**Authors:** Lais Pereira, Fabiano Oliveira, Shannon Townsend, Sonia Metangmo, Claudio Meneses, Ian N. Moore, Claudia I. Brodskyn, Jesus G. Valenzuela, Stefan Magez, Shaden Kamhawi

**Affiliations:** ^1^Vector Molecular Biology Section, Laboratory of Malaria and Vector Research, National Institute of Allergy and Infectious Diseases, National Institutes of Health, Rockville, MD, United States; ^2^Infectious Disease Pathogenesis Section, Comparative Medicine Branch, National Institute of Allergy and Infectious Diseases, National Institutes of Health, Rockville, MD, United States; ^3^Laboratorio da interação parasita hospedeito e epidemiologia, Instituto de Pesquisas Gonçalo Moniz, FIOCRUZ, Salvador, Brazil; ^4^Laboratory for Cellular and Molecular Immunology, Vrije Universiteit Brussel, Brussels, Belgium; ^5^Ghent University Global Campus, Incheon, South Korea

**Keywords:** *Leishmania major*, *Trypanosoma brucei*, coinfection, inflammation, cellular immunity, humoral immunity, cutaneous leishmaniasis, protection

## Abstract

Infection with certain bacteria, parasites, and viruses alters the host immune system to *Leishmania major* influencing disease outcome. Here, we determined the outcome of a chronic infection with *Trypanosoma brucei brucei* on cutaneous leishmaniasis (CL) caused by *L. major*. C57BL/6 mice infected with *T. b. brucei* were given a sub-curative treatment with diminazene aceturate then coinfected with *L. major* by vector bites. Our results revealed that infection with *T. b. brucei* controls CL pathology. Compared to controls, coinfected mice showed a significant decrease in lesion size (*P* < 0.05) up to 6 weeks post-infection and a significant decrease in parasite burden (*P* < 0.0001) at 3 weeks post-infection. Protection against *L. major* resulted from a non-specific activation of T cells by trypanosomes. This induced a strong immune response characterized by IFN-γ production at the site of bites and systemically, creating a hostile inflammatory environment for *L. major* parasites and conferring protection from CL.

## Introduction

Cutaneous leishmaniasis (CL) is an infectious disease caused by *Leishmania* parasites. CL affects man and other mammals, causing ulcers in the skin and mucous membranes. With a million cases registered worldwide in the last 5 years, CL is considered as a serious public health problem concentrated in poor regions of the world (1). *Leishmania* parasites are transmitted by the bite of vector sand flies together with vector-derived factors as part of a virulent infectious inoculum (2–4).

Protective adaptive immunity to CL depends on the induction of specific Th1-polarized CD4^+^T cells that produce pro-inflammatory cytokines such as IL-12, IFN-γ, and TNF-α, responsible for macrophage activation and parasite killing (5). In contrast, a Th-2 polarized immune response with T cells producing cytokines such as IL-13, IL-4, IL-10, and IL-5 are associated to susceptibility to *Leishmania* and an increase in the size and severity of *L. major* lesions (5).

Humans and animals are exposed to different species of fungi, bacteria, viruses, and parasites throughout their lifetime where the risk of co-infections is likely. Coinfections with *Leishmania* parasites and various pathogens have been reported for both humans and animals (6–12). Experimentally, studies have shown varying effects of co-infections on the progression of leishmaniasis. Mice infected with *Listeria monocytogenes* and later coinfected with *L. major* exhibited an enhanced inflammatory response and developed larger lesions compared to animals infected with *L. major* alone with no effect on parasite loads (13). Similarly, mice infected with Lymphocytic Choriomeningitis virus and subsequently coinfected with *L. major* also presented bigger lesions associated with a decrease in IFN-γ production. Coinfection with *Schistosoma mansoni* and *L. major* demonstrated that the former delays the resolution of CL in mice by decreasing the production of IFN-γ, TNF-α, and NO, and increasing IL-4 levels (14). These reports point to the importance of coinfections in modulating the outcome of CL.

Here, we demonstrate the protective effect of a chronic drug-controlled infection with *Trypanosoma b. brucei* on *L. major* infection. We show that *T. b. brucei* parasites create a non-specific intense pro-inflammatory response, local, and systemic, characterized by high levels of IFN-γ that creates an adverse environment for *Leishmania* parasites.

## Materials and Methods

### Animals and Ethics Statement

C57BL/6 mice were purchased from Charles River Laboratories (Wilmington, MA) and were housed under pathogen-free conditions at the NIAID Twinbrook animal facility in Rockville, Maryland. Animal experimental procedures were reviewed and approved by the Care and Use Committee of the National Institute of Allergy and Infectious Diseases under animal protocol LMVR4E. The Animal Care and Use program at NIAID DIR complies with the Guide for the Care and Use of Laboratory Animals and with the NIH OACU and ARAC guidelines.

### Infection of Mice With *T. b. brucei*

*Trypanosoma brucei brucei* (Antat 1.1) blood form parasites originally obtained from the Laboratory for Cellular and Molecular Immunology, Vrije Universiteit Brussel, Pleinlaan 2, 1050 Brussels, Belgium, were passaged at the laboratory of Professor S. Black, Department of Veretrinary and Animal Sciences, UMASS Amherst. Parasites were provided as frozen blood stabilates and were maintained at −70°C. *T. b. brucei* parasites were thawed and suspended in one ml of RPMI (Sigma-Aldrich) and counted in a Neubauer chamber. For infection of mice, 100 μL containing 5,000 *T. b. brucei* were injected intraperitoneally. Soluble VSG of *T. b. brucei* AnTat was prepared as described earlier (15). Throughout the study, two experimental groups were compared, mice infected with *T. b. brucei* then co-infected with *L. major* and mice infected only with *L. major*.

### Treatment of Mice With Diminazene Aceturate

Mice infected with *T. b. brucei* were treated twice at 10-day intervals with a subcurative dose of 40 mg/kg of diminazene aceturate (Berenil, Sigma-Aldrich) in 100 μL of Phosphate Buffered Saline (PBS) to control the number of systemic *T. b. brucei* parasites prior to natural transmission of *L. major* (16). The number of trypanosomes circulating in the blood were monitored at 4, 18, and 51 days after infection. Blood (2.5 μl) was collected by tail bleeds and diluted in 500 μL of phosphate-buffered saline (PBS) for counting.

### Sand Fly Infection and Transmission of *L. major* to *T. b. brucei* Infected Mice

*Lutzomyia longipalpis* sand flies, Jacobina strain, were obtained from a colony maintained at the Laboratory of Malaria and Vector Research, NIAID, NIH. Frozen amastigotes of *Leishmania major* (WR 2885) were thawed and washed, and viable amastigotes were counted in a Neubauer chamber. Artificial infection of *Lu. longipalpis* with 5 × 10^6^ *L. major* amastigotes/ml of defibrinated rabbit blood was performed as described previously (17). Eight to ten days after infection, 10 sand flies were placed in a meshed vial that was applied to a mouse ear for 2 h in the dark using custom-made clamps. Transmission of *L. major* by bites of infected sand flies was performed on day 30 after infection with *T. b. brucei* and 10 days after the second treatment with diminazene aceturate.

### Measurement of *L. major* Lesion Size

The diameter(s) and thickness(s) of the developing *L. major* lesions were measured at 2, 4, and 6 weeks after exposure to infected *Lu. longipalpis* sand flies using a Vernier caliper (Mitutoyo, Baltimore, MD). The sum of the area of the developing lesions was used to assess disease progression.

### Elisa

IgG titers against *L. major* were measured using *L. major* cell lysate antigen (Leish). ELISA plates (Immulon4-Thermo, Waltham, MA) were coated overnight 4°C with 20 μg of Leish/ml bicarbonate carbonate buffer pH 9.6. After washing and blocking with 4% BSA for 2 h/37°C, 50 μL of serum (1:100) were incubated for 1 h at 37°C. After washing, plates were incubated with alkaline phosphatase–conjugated anti-mouse IgG (1/1000, BD Biosciences). The reaction was revealed using alkaline phosphate substrate (Promega) and absorbance was read at 405 nm.

Briefly, to measure antibodies against *T. b. brucei* or *Brugia malayi* wells were coated overnight with 10 μg of either *T. b. brucei* AnTat 1.1 (cell lysate) or AnTat 1.1 sVSG (a purified surface membrane protein), or 10 μg of *Brugia malayi* microfilariae cell lysate. After washing and blocking, mice sera (1:50) were incubated for 2 h at 37°C. After washing, plates were incubated with alkaline phosphatase–conjugated anti-mouse IgG (1/1000, BD Biosciences). The reaction was revealed using alkaline phosphate substrate (Promega). Absorbance was read at 405 nm.

### *L. major* Load by Limiting Dilution Assay (LDA)

The lymph node draining the infected ear was macerated and homogenized in 200 μl Schneider's (Gibco, NY) supplemented with 10% heat inactivated fetal bovine serum (Gibco, NY), 2 mM L-glutamine,100 U/ml Penicillin and 100/ml Streptomycin (complete Schneider medium). The macerate was serially diluted (1:2) in 96-well flat bottom microtiter plates containing 50 ml biphasic medium prepared using NNN medium with 10% of defibrinated rabbit blood overlaid with 200 μl complete Schneider medium. The plates were incubated at 27°C and examined up to 10 days after culture. The number of live *L. major* promastigotes was determined from the highest dilution at which *L. major* could be grown.

### Evaluation of Brain Histopathology in *T. b. brucei*-Infected Mice

Each brain section was fixed in 10% neutral buffered formalin for 24 h, processed and sectioned (5 mm), and stained with hematoxylin-eosin (Histoserv, Germantown, MD). Slides were evaluated by a board-certified veterinary pathologist and photomicrographs were taken using the Olympus BX51 microscope and Olympus DP73 camera.

### *In vitro* Stimulation of Spleen Cells With *L. major* Cell Lysate (Leish)

Spleens were pooled by group from 5 to 7 mice for flow cytometry. The spleens were macerated and treated with ACK Lysis buffer (Lyfe technologies, USA) for 5 min to lyse erythrocytes. After washing, 5 × 10^6^ splenic cells/mL were cultured in RPMI medium (Life Technologies) supplemented with 10% heat-inactivated fetal bovine serum, 100 U/ml of penicillin, 100 mg/ml of streptomycin (Sigma, St. Louis, MO) in the presence of 50 μg/ml of Leish. Cells were incubated at 37°C with 5% CO_2_ for 24 h.

### Flow Cytometry

Non-specific binding sites on viable splenic cells were blocked for 10 min at 4°C, using anti-CD16/32 FcγR antibody (BD). After washing, the cells were incubated with a Life/Dead stain (Life Technologies) for 20 min to exclude dead cells from the analysis. T cells and cytokines were identified using the following anti-mouse antibodies: PerCP- labeled anti-CD4, APC-Cy7 labeled anti-TCR-β, APC labeled anti-IFN-γ, and Pacific Blue labeled anti-IL10. For B cell staining the following anti-mouse antibodies were used: PE labeled anti-CD138 and APC labeled anti-B220. Incubation with all antibodies were conducted for 30 min at 4°C. All samples were acquired using a MACSQuant (Miltenyi Biotec) and data were analyzed with the FlowJo V10 software package.

### RNA Extraction and cDNA Synthesis

Total RNA was isolated from the ear of each mouse using the Ambion Kit (Life Technologies) according to the manufacturer's instructions. cDNA was obtained using 100 μg RNA from individual mice ears that was synthetized with 4 μL de qScript cDNA (SuperMix Superscript III, Invitrogen) following the manufacturer's instructions. The cDNAs were stored at −20°C until cytokine analysis by Real time PCR.

### Real Time PCR for Cytokine Quantification

PCR was carried out using the Perfect Master mix (Roche Diagnostics) and gene specific primer sets for IFN-γ, TNF-α, IL-10, IL-12, and IL-4 using the Light Cycler 480 (Roche Diagnostics). A standard curve for each set of primers was generated as recommended by the manufacturer. The expression levels of the genes of interest were normalized to RNA levels of GAPDH, an endogenous gene. The results are expressed in fold change over gene expression in the control group.

### Statistical Analysis

Graphs and statistical significance were prepared and analyzed using GraphPad Prism Software 7.0. An unpaired *t*-test followed by Mann-Whitney test or a one-way ANOVA followed by Tukey's multiple comparisons test were used to evaluate statistical significance among groups. A *p* value < 0.05 was considered statistically significant.

## Results

### A Treatment Regimen With Diminazene Aceturate Produces a Chronic Infection With *T. b. brucei*

**Figure 1:**
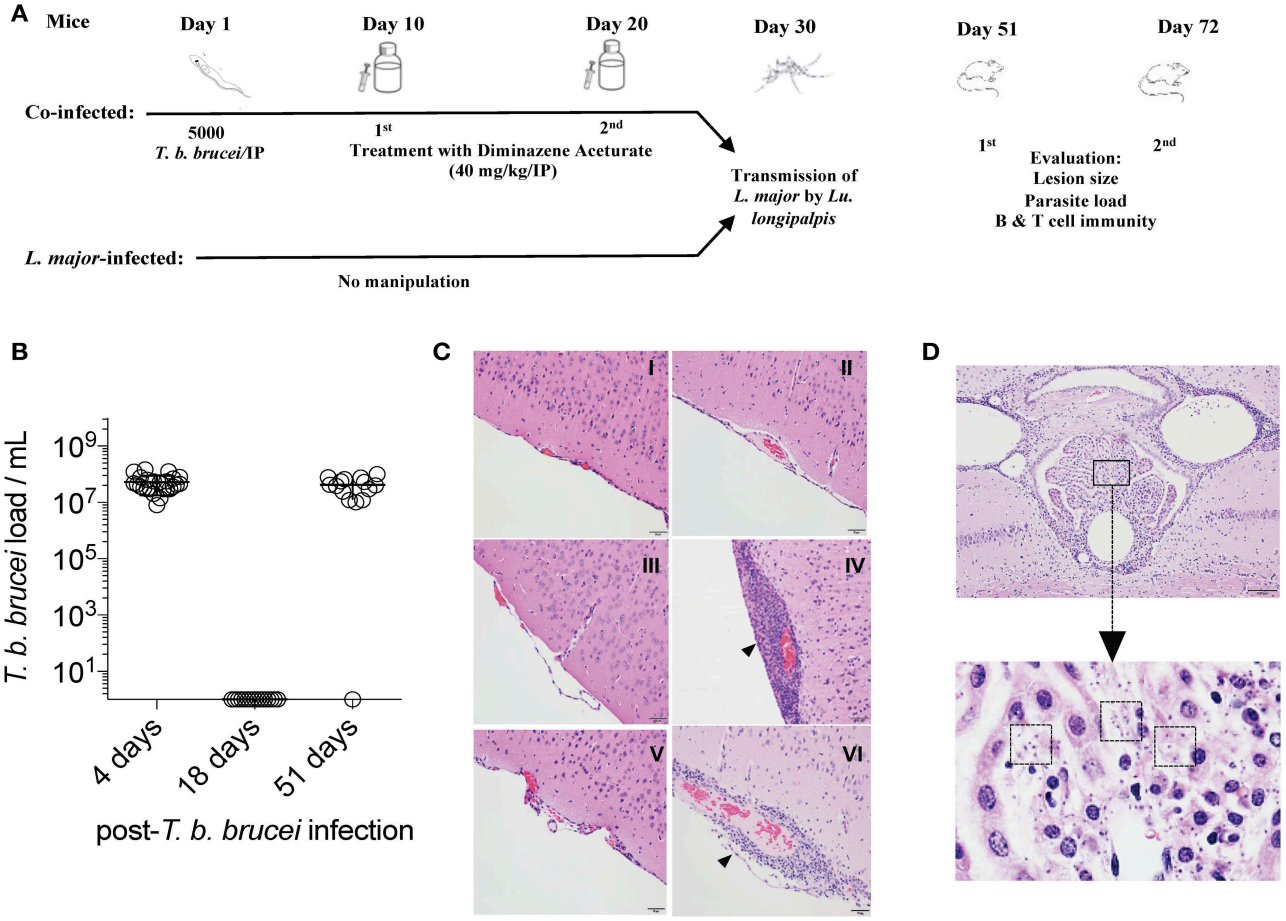
A model for a drug-controlled chronic infection with *T. b. brucei*. **(A)** A schematic representation of the experimental study design. Drug, diminazene aceturate. **(B)** *T. b. brucei* parasite load per ml of blood in mice before the first and second drug treatments (days 4 and 18, respectively), and 3 weeks after exposure to 10 sand flies infected with *L. major* (day 51). Data are representative of three independent experiments (*n* = 8–13 mice). **(C,D)** Brain sections of mice stained with hematoxylin and eosin. **(C)** naive (I) and 10 days after infection with *T. b. brucei* (II), and 3 or 6 weeks after infection with *L. major* in the absence (III, V) or presence of *T. b. brucei* (IV, VI), respectively. Arrows highlight areas of inflammation. Scale, 50 μm. **(D)** Magnification of the choroid plexus (lower panel) within the dorsal third ventricle of the brain (upper panel) 6 weeks after infection with *L. major* in the presence of *T. b. brucei*. Dashed boxes highlight areas with *T. b. brucei* parasites. Scale, 100 μm). Pictures are representative of two independent experiments (*n* = 5 mice/group).

Treatment of mice with two doses of 40 mg/kg diminazene aceturate at 10-day intervals after the intraperitoneal injection of 5,000 *T. b. brucei* (Figure 1A) maintained a chronic drug-controlled infection with *T. b. brucei* (Figure 1B). We chose three timepoints to assess the number of circulating blood trypanosomes: Four days, before the first drug treatment; 18 days, before the second drug treatment; and 51 days, at the first evaluation after *L. major* infection. Counts of *T. b. brucei* circulating in the blood demonstrated the growth of parasites on day four, prior to drug treatment. On day 18, two days before the second drug treatment, the number of *T. b. brucei* parasites declined to sub-detectable levels in blood indicating their transient clearance from circulation (Figure 1B). The persistence of *T. b. brucei* parasites in co-infected mice after the second drug treatment was confirmed by their recovery to pre-drug treatment levels on day 51, three weeks after *L. major* infection (Figure 1B).

To investigate the consequence of the persistence of *T. b. brucei* after treatment with two doses of 40mg/kg diminazene aceturate, histopathologic analyses of the brain of mice was conducted 10 days after *T. b. brucei* infection, and three and 6 weeks after transmission of *L. major* to mice (Figure 1C). Ten days after infection with 5,000 *T. b. brucei* and prior to the first dose of drug, the brains from infected and naive mice, the latter representing a steady state baseline, were similar and unaltered (Figure 1C, I and II). In comparison, three weeks after infection with *L. major* (51 days after infection with *T. b. brucei* and 31 days after administration of the second drug dose), the brains of coinfected mice demonstrated acute meningoencephalitis that was absent from mice infected with *L. major* alone (Figure 1C, III and IV). The meningoencephalitis in brains of coinfected mice was characterized by an intense neutrophilic infiltrate within the meninges, extending at a lesser degree to different regions of the neuroparenchyma (Figure 1C, IV). Importantly, we observed a few *T. b. brucei* parasites in the choroid plexus of the third ventricle, indicating that the blood/brain barrier has been crossed. At six weeks after infection with *L. major* (72 days after infection *T. b. brucei* and 52 days after administration of the second drug dose) the brains of mice infected with *L. major* alone were unchanged while the brain tissue of coinfected mice showed a more chronic meningoencephalitis that was composed largely of lymphocytes and plasma cells (Figure 1C, V and VI). Additionally, more *T. b. brucei* parasites were evident (Figure 1D, Dashed boxes) within the choroid plexus of the third ventricle (Figure 1D) which also displayed a large number of plasma cells. Of note, mice coinfected with *T. b. brucei* and *L. major* also exhibited a lower rate of weight gain beyond the second week after *L. major* transmission compared to animals infected with *L. major* alone (Supplementary Figure 1).

### *T. b. brucei* Infection Results in a Sustained Control of *Leishmania major* Lesion Pathology Following Vector-Transmission

**Figure 2:**
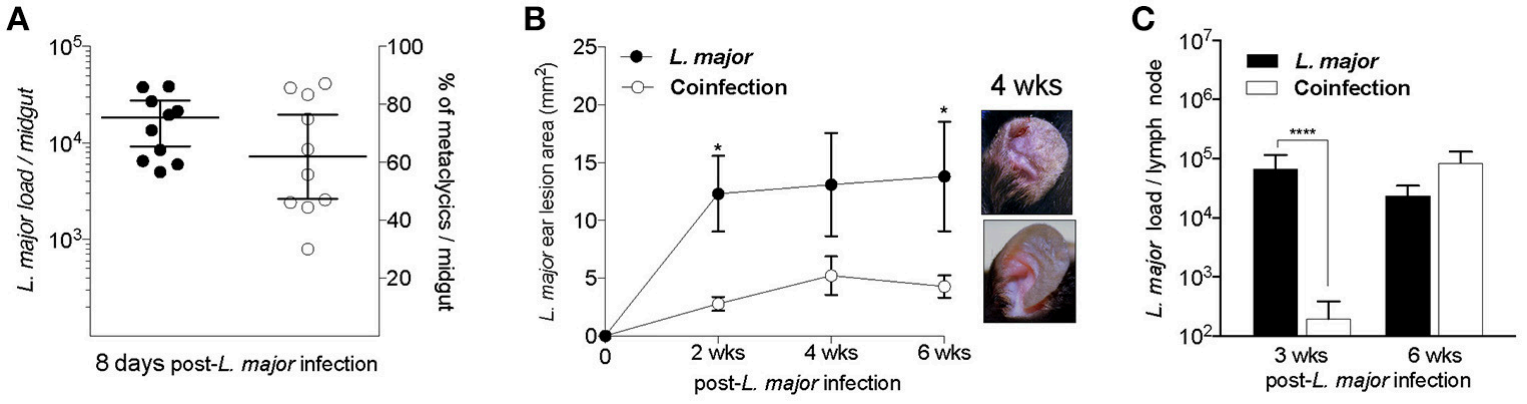
A chronic infection with *T. b. brucei* controls *L. major* lesion pathology. **(A)** The parasite burden and percent metacyclics in *Lu. longipalpis* sand flies at the time of *L. major* vector-transmission to mice. **(B,C)** Mice infected with *L. major* by vector challenge in the absence (*L. major*) or presence (Coinfection) of a preceding infection with *T. b. brucei*. **(B)** Area of *L. major* ear lesions. Pictures show the pathology of cutaneous lesions at 4 weeks after *L. major* infection. *C, L. major* parasite load at 3 and 6 weeks after infection. ^*^P < 0.05; ^****^P < 0.0001; Unpaired two-tailed *t*-test. Data are representative of three independent experiments (*n* = 8–13 mice).

To determine the influence of a *T. b. brucei* infection on the development of CL, mice infected or not with *T. b. brucei* were challenged 10 days after the second dose of diminazene aceturate with 10 *L. major*-infected *Lu. longipalpis* sand flies harboring mature infections with a geometric mean parasite load of 10^4^ and an average of 60% infectious metacyclics per midgut (Figure 2A). After transmission, we followed the course of developing ulcers by measuring the lesion(s) area in mice ears. Mice coinfected with *T. b. brucei* and *L. major* developed significantly smaller lesions (*P* < 0.05) up to 6 weeks post infection with *L. major* (Figure 2B) and did not manifest open ulcers (Figure 2B, pictures) compared to control mice infected with *L. major* alone. Interestingly, a significant reduction in *Leishmania* parasite ear burden was observed in coinfected compared to controls at 3 weeks post-infection, however, by 6 weeks the parasite number in coinfected mice recovered in the absence of pathology and were comparable in number in both groups (Figure 2C).

### *T. b. brucei* Infection Induces Expansion of the Splenic Plasma B Cell Compartment

**Figure 3:**
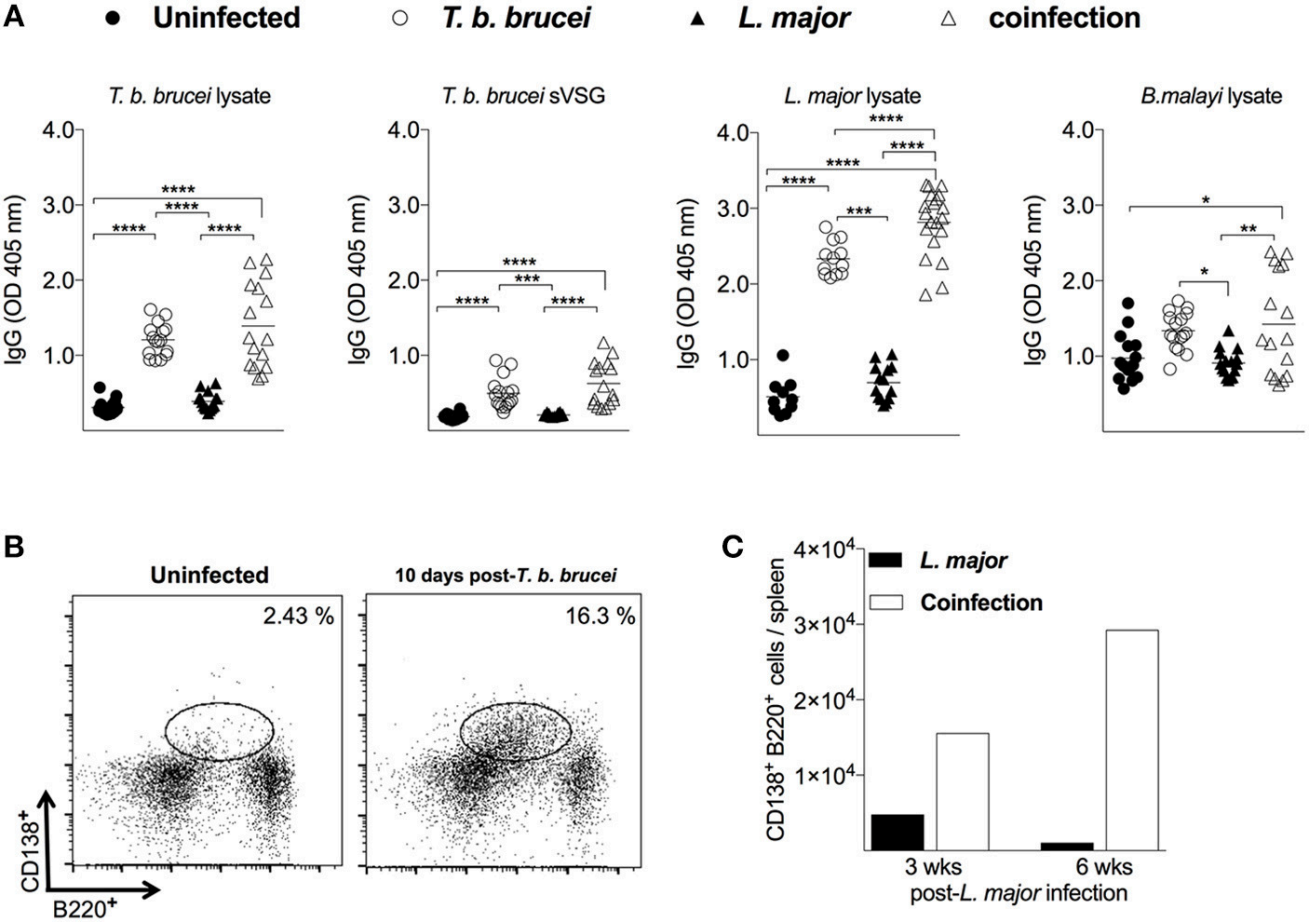
The humoral immune response in mice coinfected with *T. b. brucei* and *L. major*. Mice serum and spleen cells were collected from naive animals, 10 days after infection with *T. b. brucei*, and 3 and 6 weeks after infection with *L. major*. **(A)** Total IgG levels against *T. b. brucei* AnTat 1.1 cell lysate, *T. b. brucei* AnTat 1.1 soluble VSG, *L. major* cell lysate, or *B. malayi* microfilaria cell lysate. **(B)** The frequency of splenic B220^+^/CD138^+^ plasma cells before and 10 days after infection with *T. b. brucei*. Cell frequency is indicated on the upper left corner of dot plots. **(C)** The absolute number of plasma cells in pooled spleens of mice at 3 and 6 weeks following challenge with *L. major*-infected sand flies. *T. b. brucei*, infection with *T. b. brucei* alone; *L. major*, infection with *L. major* alone; Coinfection, infection with *L. major* following a preceding infection with *T. b. brucei*. ^*^P < 0.05; ^**^P < 0.01; ^***^P < 0.001; ^****^P < 0.0001, one-way ANOVA followed by Tukey's multiple comparisons test. Representative data from 2 to 3 independent experiments are shown. (**A**, *n* = 10–17 mice; **B,C**, *n* = 5–7 mice).

To investigate whether the reduced *L. major* pathology in coinfected mice is associated to cross-reactive immunity directed against similar trypanosomatid antigens, we tested the serum from mice infected with *L. major* in the presence or absence of *T. b. brucei* against the cell lysate or soluble VSG protein from the trypanosomes. Animals infected with *L. major* alone had no cross-reactive antibodies to either anti-AnTat 1.1 Lysate or VSG (Figure 3A), suggestive of the absence of shared antigens. Conversely, mice coinfected with both *T. b. brucei* and *L. major* reacted to both antigens (*P* < 0.0001, Figure 3A). Counterintuitively, 10 days after infection with *T. b. brucei*, and before infection with *L. major*, mice showed significantly higher IgG titers against *L. major* cell lysate (Figure 3A, *P* < 0.0001) as well as the cell lysate from an unrelated parasite, *Brugia malayi* microfilaria (Figure 3A, *P* < 0.01), compared to naive mice indicative of polyclonal activation of B cells in trypanosome-infected mice. Of note, a further increase in the IgG titer against *L. major* cell lysate was observed three weeks after coinfection with *L. major* (Figure 3A).

To assess the activation state of B cells in *T. b. brucei* infected mice, a plasma cell specific B220^+^/CD138^+^ staining was performed on spleen cells. Ten days following infection with *T. b. brucei*, there was a seven-fold increase in plasma B cells compared to the basal state of naive mice (Figure 3B). Moreover, at 3 and 6 weeks after transmission with *L. major*-infected sand flies a 3- and 28-fold expansion of plasma cells was observed in coinfected mice compared to animals infected with *L. major* alone (Figure 3C), likely due to the increasing number of trypanosomes in circulation.

### *T. b. brucei* Infection Produces an Inflammatory Environment at the Site of *L. major-*Infected Vector Bites and Systemically

**Figure 4:**
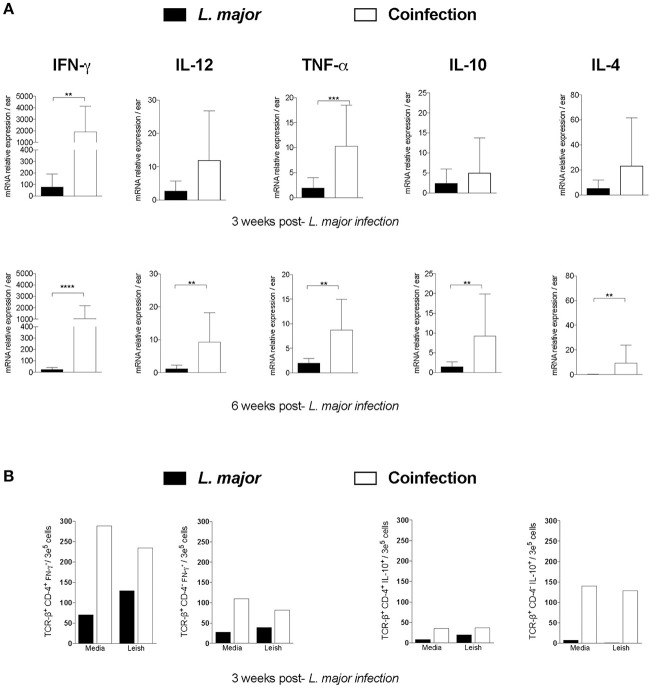
The cellular inflammatory response in mice after infection with *L. major* in the presence of absence of *T. b. brucei*. Mice infected or not with *T. b. brucei* were exposed to 10 *L. major*-infected *Lu. longipalpis* sand flies. **(A)** The local inflammatory response in mice ears at 3 and 6 weeks after infection with *L. major*. mRNA expression relative to naive mice ears was determined for IFN-γ, TNF-α, IL-12, IL-4, and IL-10 by RT-PCR. Cumulative data from 2 independent experiments are shown (*n* = 8–15 mice). Bars denote the mean ± 1SD. *T. b. brucei*, infection with *T. b. brucei* alone; *L. major*, infection with *L. major* alone; Coinfection, infection with *L. major* after a preceding infection with *T. b. brucei*. ^**^P < 0.01; ^***^P < 0.001; ^****^P < 0.0001, unpaired two-tailed *t*-test. **(B)** The number of CD4^+^ and CD4^−^ T cells producing IFN-γ or IL-10 from mice spleens three weeks after vector-transmission of *L. major* to mice infected or not with *T. b. brucei*. Pooled spleen cells were left unstimulated (Media) or were stimulated with *L. major* cell lysate (Leish). Representative data from 2 independent experiments are shown (*n* = 8 mice).

To understand the basis of the protection from *L. major* conferred by an active infection with *T. b. brucei*, we investigated the *ex vivo* expression of cytokines in mice ears 3 and 6 weeks after exposure to infected sand fly bites. Three weeks after the bites of *L. major*-infected sand flies, the ears of mice that were also infected with *T. b. brucei* showed a significant increase in the local expression of the pro-inflammatory cytokines IFN-γ (*P* < 0.01) and TNF-α (*P* < 0.001) when compared to mice infected with *L. major* alone (Figure 4A). Importantly, six weeks after *L. major* infection, the expression of both pro- and anti-inflammatory cytokines was significantly enhanced (*P* < 0.01) in coinfected compared to controls though the local milieu was still dominated by IFN-γ (Figure 4A). This indicates that *T. b. brucei* induces a cell-mediated as well as a humoral hyper-inflammatory environment.

The induction of a cellular hyper-inflammatory environment after infection with *T. b. brucei* was systemic. At three weeks post-infection with *L. major*, the number of IFN-γ-producing, and to a lesser extent IL-10-producing, TCRß^+^CD4^+^ splenic T cells (Supplementary Figure 2) was higher in mice coinfected with *T. b. brucei* compared to mice only infected with *L. major*, with or without stimulation with *L. major* cell lysate (Leish), emphasizing the pro-inflammatory nature of the response (Figure 4B). TCRß^+^CD4^−^ splenic T cells (Supplementary Figure 2), that include CD8^+^ T cells, of mice coinfected with *L. major* and *T. b. brucei* also produced higher levels of IFN-γ and IL-10 compared to mice only infected with *L. major*, but consisted mainly of IL-10-producing cells (Figure 4B). Interestingly, in the absence of antigen stimulation, the number of CD4^+^ and CD4^−^ T cells producing IFN-γ were about 4-fold higher in co-infected compared to *L. major*-infected mice, and were about 2-fold higher after stimulation with Leish.

## Discussion

Coinfections in humans are more the norm than the exception, particularly in developing countries where infectious diseases are prevalent (18–20). For *Leishmania* parasites, coinfection with HIV is the best characterized. HIV enhances susceptibility to *Leishmania* reinfection and relapse, increases lethality of VL, and influences disease prevalence worldwide threatening control and elimination efforts [www.who.int/leishmaniasis/burden/hiv_coinfection/burden_hiv_coinfection/en/; (21–24)]. Experimentally, several models of coinfection with *Leishmania* also resulted in disease enhancement (13, 14). Here, we demonstrate that infection of mice with *T. b. brucei* has a protective effect on *L. major* infection despite a virulent challenge via infected sand fly bites. Using a mouse model of coinfection, we show that an infection with *T. b. brucei* affects both the humoral and cellular arms of the host immune system generating a non-specific polyclonal activated state. This creates an inflammatory environment dominated by high levels of IFN-γ that adversely affects the invading *Leishmania* parasites. Moreover, this *T. b. brucei*-induced hyperinflammatory state was observed locally in the skin at bite sites and systemically, suggesting that its protective effect may influence both visceral as well as cutaneous leishmaniasis.

Though we observed the well-established severe effect of *T. brucei* on the B cell compartment, with infection inducing non-specific polyclonal B cell activation with production of nonspecific antibodies, B cell apoptosis, and loss of memory B cells (25, 26), it is well established that the humoral response is of little importance in protective immunity to *L. major* (27, 28). Instead, protection is mainly conferred by IFN-γ-producing CD4 T cells (5, 29). Excessive production of cytokines is a hallmark of African trypanosome infections (30–32). Moreover, IFN-γ production by variant surface glycoprotein-specific CD4 T cells has been known to be important for both control of (early stages), and susceptibility to (late stages), infection with African trypanosomiasis (31, 32). Here, mice coinfected with *T. b. brucei* and *L. major* exhibited a strong inflammatory response in *L. major*-infected mice ears that was dominated by high levels of IFN-γ. Though other cells such as NK cells could have contributed to the large amounts of IFN-γ produced in the skin, the robust IFN-γ response of CD4 T cells in the spleen indicates that this cell population is likely a major contributor to the proinflmmaotry environment in the ear 3 weeks post infection with *L. major*. This inflammatory response was sustained throughout the study timeline and was significantly higher compared to mice infected with *L. major* alone elucidating the mechanism underlying protection against CL pathology in trypanosome-infected mice. Moreover, TCRß^+^CD4^−^ splenic T cells, that include CD8^+^ T cells, also participated to a lesser extent in the inflammatory response caused by *T. b. brucei* supporting previous findings in a *T. b. brucei* AnTat 1.1/C57BL6 mice model of infection where CD8 T cells were implicated in IFN-γ production (32).

In mice, infection with *T. brucei* species can be cured if diminazene aceturate is given early, within 3–7 days after infection (33). However, if given later or at suboptimal drug doses, trypanosomes can infect and proliferate in the brain where they are protected by the blood brain barrier from the effect of drugs, becoming a permanent source of relapse (16, 33). In this study, two doses of 40 mg/kg of diminazene aceturate given at 10 and 20 days after injection with 5,000 *T. b. brucei* AnTat 1.1 resulted in a chronic drug-controlled infection with brain inflammation observed at 51 and 71 days post-trypanosome infection. As such, this model can be adapted for the study of chronic trypanosome infections. A similar model using a drug regimen with Moranyl has been successfully used to establish a chronic *T. b. brucei* AnTat 1.1 infection where mice develop meningoencephalitis (34). Developing chronic models of ongoing trypanosome infections would be useful for the study of coinfections requiring extended study time lines.

Both leishmaniasis and Human African trypanosomiasis (HAT) are vector-borne diseases transmitted by sand flies and tsetse flies, respectively (35, 36). Generally, climate change, conflict and globalization have promoted the spread of vector-borne diseases (1, 37). For leishmaniasis and HAT, models have predicted expansion of their vector ranges due to climate change (37, 38). Combined with continued conflict and population displacement, this will likely increase the areas where both diseases co-exist increasing the chances of coinfections. At present, leishmaniasis is more broadly distributed while HAT remains restricted to sub-Saharan Africa (39, 40). Interestingly, despite regions where both diseases are endemic, most notably South Sudan where a high number of both *T. b. gambiense* HAT and VL cases have been reported (39, 41), to our knowledge there have been no documented human cases of HAT/VL coinfections. Potentially, HAT infected individuals may be resistant to leishmaniasis.

## Author Contributions

LP, SMa, and SK designed the study. LP, ST, SMe, and CM carried out experiments. LP, FO, IM, and SK analyzed the data. LP, FO, IM, CB, JV, SMa, and SK wrote the manuscript.

### Conflict of Interest Statement

The authors declare that the research was conducted in the absence of any commercial or financial relationships that could be construed as a potential conflict of interest.
