# Supplementary material for: Coinfection With Trypanosoma brucei Confers Protection Against Cutaneous Leishmaniasis
Source: Front Immunol. 2018 Dec 11;9:2855. doi: 10.3389/fimmu.2018.02855 (PMC6297747; doi:10.3389/fimmu.2018.02855)
Supplement: Supplementary file 1 [file Data_Sheet_1.PDF]

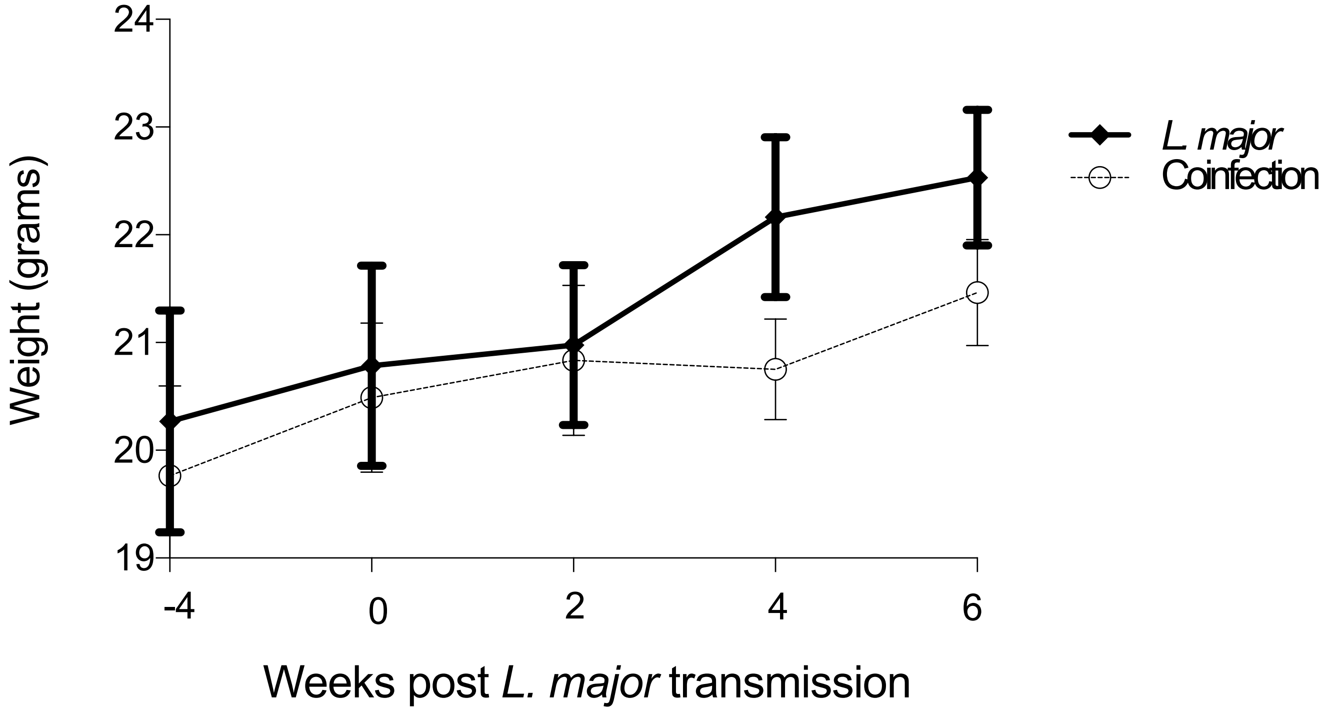

Supplementary Figure 1. Body weight of mice infected with *L. major* in the presence or absence of *T. b. brucei*. *L. major*, infection with *L. major* alone; Coinfection, infection with *L. major* following a preceding infection with *T. b. brucei*. Cumulative data from 2 independent experiments are shown; n= 15-30 mice.

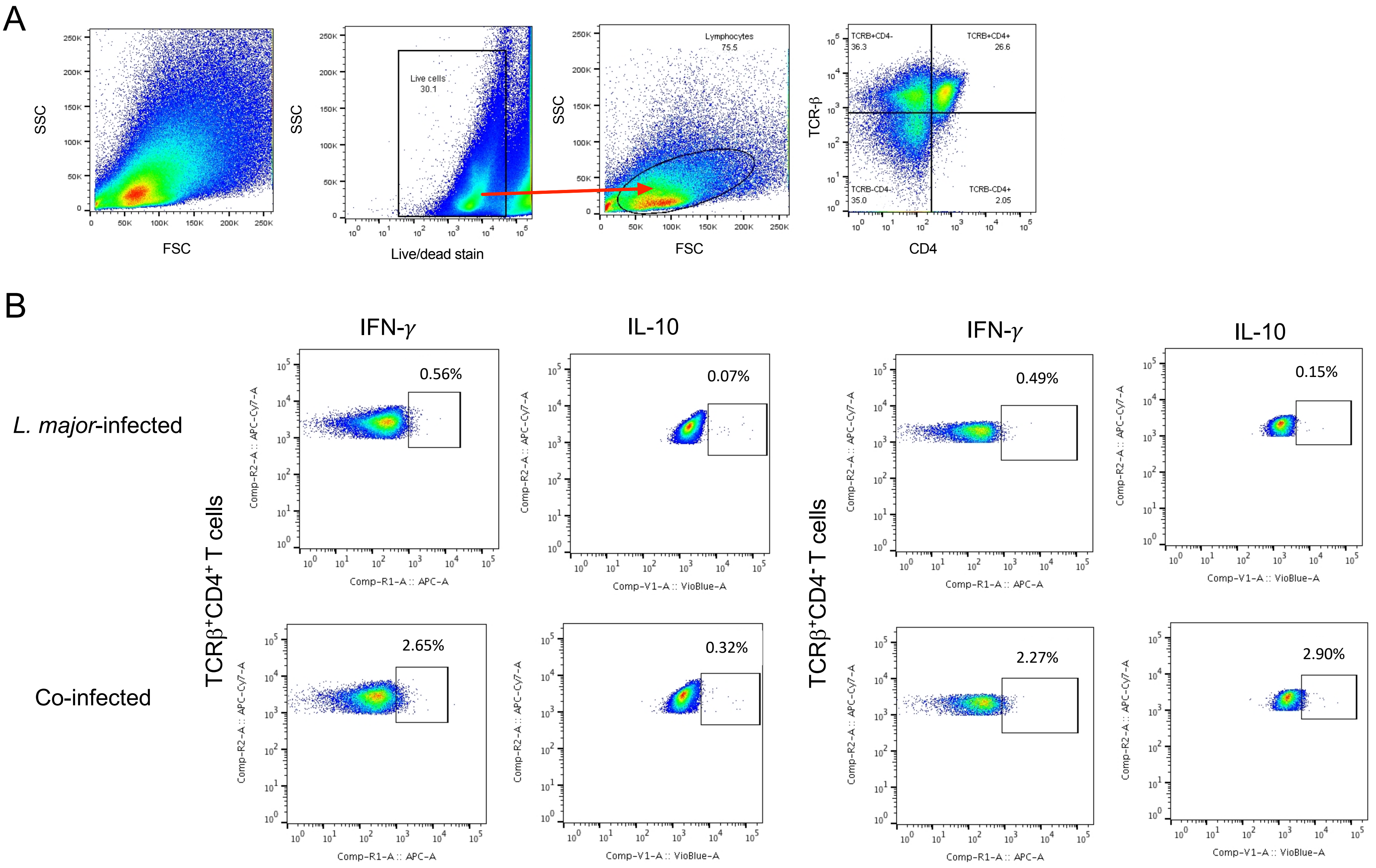

Supplementary Figure 2. Flow cytometry of pooled mice spleen cells. A, Gating strategy. Live cells were gated on TCRβ$^+$CD4$^+$ and TCRβ$^+$CD4$^-$ populations. B, representative plots of unstimulated CD4$^+$ and CD4$^-$ T cells producing IFN-γ or IL-10 three weeks after vector-transmission of *L. major* to mice infected with *T. b. brucei* (co-infected) or not (*L. major*-infected).
